# A Qualitative Thematic Analysis of Addressing the Why: An Artificial Intelligence (AI) in Healthcare Symposium

**DOI:** 10.7759/cureus.23704

**Published:** 2022-03-31

**Authors:** Joshua T Borgstadt, Edward A Kalpas, Hayden M Pond

**Affiliations:** 1 Medical Science, A.T. Still University, Phoenix, USA; 2 Clinical Informatics, Academic Affairs, HonorHealth and College of Health Solutions, Arizona State University, Scottsdale, USA; 3 Research, HonorHealth, Scottsdale, USA

**Keywords:** artificial intelligence in medicine, bias in healthcare, transparency, workflow augmentation, knowledge augmentation, interoperability

## Abstract

Healthcare managers and clinicians are inefficient in the processes of workflows and documentation. The inefficiency is due in part by increasing demands of insurance companies, regulatory demands from the government, and human error. Artificial intelligence (AI) can improve healthcare processes by decreasing variability, thus improving patient and physician experience and patient outcomes. This project brings together a panel of five experts to discuss problems in medicine and some of the tools available through AI and technology to address these problems. The symposium modeled a "flipped classroom" format. The first five 20-minute modules were uploaded to a web-based platform for viewing in advance of the 60-minute moderated roundtable (Zoom, Zoom Video Communications, San Jose, CA, USA). The following themes emerged after reviewing the transcribed data: data privacy and access (N=3, number of times identified); process improvement (N=2); physician experience (N=1); value in data (N=2); and bias in healthcare and AI (N=3). For AI to become implemented on a large scale in healthcare, many areas will need continued discussion and research, including a continued look into how AI can add value to workflow and knowledge augmentation. In addition, standards for the implementation of AI and a methodical approach to the analysis of the effectiveness of algorithms coupled with training of healthcare professionals in the language of AI algorithms will be helpful to ensure that AI is integrated safely.

## Introduction

According to a report by Johns Hopkins, medical errors are now the third leading cause of death behind cardiovascular disease and cancer [1]. The study details inefficient processes and distracted and inconsistent care as causative factors, not bad doctors. Medicine is a profoundly personal profession, especially in primary care. Providers take care of patients from the womb to the tomb and everything in between. A patient has an expectation for their primary care provider to be empathetic and knowledgeable in their craft. Instead, individuals often encounter burned-out providers, overburdened by inefficient documentation within electronic medical records, inefficient processes, and inadequate clinic staffing [2]. The pandemic has highlighted the importance of adequate staff, the mental and physical health of staff, and an efficient process for a health system to meet the growing demands of the public. Often, the realities of a complex system that cannot function at the highest level loom large over the reality of the public in desperate need of the proper care at the right time.

The 2021 update of the Commonwealth Fund, which looks into health outcomes among high-income countries, does not cast a favorable view of the United States healthcare system [3]. The report looked at 71 measures across five areas: access to care, care process, administrative efficiency, equity, and health outcomes [3]. The United States was last overall. The United States came in second on measuring care processes; however, it ranked last on the remaining four measures [3]. This rank is in stark contrast to the number of dollars spent on healthcare in the United States. The United States far outspends the other countries regarding the percentage of gross domestic product on related healthcare dollars [3]. Artificial intelligence (AI) is gaining attention as a disruptor of the status quo in medicine. Great promise and potential lie within AI as a growth agency to improve process efficiency and care within medicine.

AI is considered by many the most recent industrial revolution, detailed in an article in Forbes entitled, "The 4th Industrial Revolution Is Here, Are You Ready?" [4]. AI has revolutionized the way we communicate and interact with the supply chain and has increased efficiency in multiple industries, ultimately increasing profit margins. According to a white paper from Accenture, AI can increase healthcare profits by 55% by the year 2035 [5]. Integration of AI into primary care is part of this growth. Currently, AI is being used and tested in specialties such as radiology, cardiology, and oncology [6,7]. Specialties that are dependent on imaging have seen the rapid acceptance of AI pilot programs due to the ability of AI to synthesize large data sets, evaluate, and accurately diagnose. Some of these AI programs are showing accuracy in diagnosis to the same degree as or better than human physicians. The appropriate application of this technology continues to be researched [6,7]. 

Healthcare organizations have begun to adopt AI systems and have successfully implemented aspects of this technology into their daily process. However, AI has yet to gain full acceptance throughout healthcare. AI has the potential to garner mainstream attention; however, it must first gain the trust of patients, providers, and staff while showing viability as a business model within clinics and health systems. 

This project looks at the themes garnered from a thematic analysis of an online symposium on AI in medicine. The objectives of the symposium include 1) current trends in AI in medicine; 2) short-term and long-term potential of AI in medicine to address issues such as patient access, patient engagement, and patient safety; and 3) understanding the current barriers to the implementation and utilization of AI in medicine. 

## Materials and methods

In June 2021, five expert speakers convened a web-based symposium to discuss some of the more controversial topics around AI. The industry experts include a data scientist from a university with research around data mining, a senior program engineer from a large electronic medical record company, an executive from a prominent AI healthcare platform, a chief medical information officer with a large local health system, and a fellow from a medical informatics program. The first five 20-minute modules were uploaded to a web-based platform for viewing in advance of the 60-minute moderated roundtable (Zoom, Zoom Video Communications, San Jose, CA, USA), modeling a "flipped classroom" curricular design. The interactive 60-minute moderated roundtable provided an opportunity for participants to engage directly with the presenters, ask questions, and critically analyze the topics in a meaningful way. The panel discussion was transcribed with three authors reviewing the themes (identified here as EK, HP, and JB). An inductive thematic analysis of a semi-structured moderated panel on AI in medicine was performed utilizing an iterative process. The transcription was reviewed multiple times, with codes from each reviewer identified. Common themes from these codes were analyzed and condensed for dissemination and included data privacy and access, process improvement, physician experience, value in data, and bias in healthcare and AI. 

For the evaluation of themes, a topical literature search was conducted utilizing Google Scholar (Google, Mountain View, CA, USA) with the following queries: AI and data privacy and data access, AI and process improvement, AI and physician's experience, AI and bias in healthcare, and AI and value in data. Articles with a published date of January 2020 to the present were considered (Table 1).

**Table 1:** Education program, title, and objectives AI: artificial intelligence.

Session title (speaker initials)	Objectives
	Principles in AI (CF)	1. Describe the clinical concepts in AI. 2. Provide examples of how AI is used in clinical practice, including ambulatory and inpatient medicine. 3. Develop the strategies to implement AI and to improve healthcare outcomes.
Bias in AI (CN)	1. Discuss the etiology of bias in healthcare data. 2. Describe the consequences of biases in data, AI, and machine learning systems. 3. Apply the strategies to acknowledge and eliminate bias in data systems.
Relationship between AI and clinician burnout (RC)	1. Review the role of administrative burden on the incidence of clinician burnout. 2. Develop an AI infrastructure that may improve both the patient and clinician experience.
Case studies in AI (EK)	1. Compare and contrast the case-based scenarios in the use of AI in clinical medicine. 2. Analyze the mechanisms for improving the integration of AI in medicine.
Process mining: how to discover processes hidden in the electronic health record (EHR) data (AG)	1. Explain what process mining is. 2. Describe the role of machine learning in process mining. 3. Give examples of how process mining can be used in healthcare. 4. Argue the strengths and drawbacks of process mining.
Moderated roundtable discussion with all presenters (JB)	1. Discuss "why we do what we do" in medicine (i.e., patient access, patient engagement, quality improvement, patient safety, medical innovation) and the role of AI in these processes. 2. Synthesize the key challenges in data and AI infrastructure for the next 5-10 years in healthcare. 3. Recommend the strategies to improve our approach to healthcare outcomes (using a data-driven approach).

## Results

The following themes emerged after reviewing the transcribed data: data privacy and access (N=3, number of times identified); process improvement (N=2); physician experience (N=1); value in data (N=2); and bias in healthcare and AI (N=3) (Table 2).

**Table 2:** Thematic analysis

Theme quantification
Data privacy and access: EK (1); HP (1); JB (1)
Bias in healthcare: EK (1); HP (1); JB (1)
Process improvement: EK (0); HP (1); JB (1)
Value of AI: EK (1); HP (0); JB (1)
Physician experience: EK (1); HP (0); JB (0)

Data from the symposium were synthesized utilizing an iterative process. The transcription was analyzed, and the section below reflects the synthesis of themes followed by quotes from presenters supporting the themes. The discussion section applies medical literature to each theme for further evaluation.

Data privacy and access

Large amounts of data exist with electronic medical records (EMRs), smartphones, and mobile devices; how do we utilize technology to synthesize data for process improvement and quality measures while maintaining patient privacy? In the United States, organizations own their data. How do we share data among health systems in a meaningful way while maintaining privacy? Is there a way to compensate patients for the use of their data? Would this incentivize patients to engage in programs that seek data for purposes of research? The appropriate analysis and dissemination of data between organizations in healthcare provide an opportunity for insight to improve care delivery. Nations with a centralized healthcare system can draw on large amounts of data without privacy concerns when sharing between organizations. The United States has a siloed system where each organization owns the data of the patient population. Interoperability is an essential piece in improving data sharing in the US healthcare system. Steps must be taken to ensure data collection, and sharing is done ethically.

One solution discussed by presenters is training in basic algorithms, data governance, and interoperability. Patients and healthcare professionals generally lack an understanding of AI and data management. Tech companies like Google and Amazon have a competitive advantage over health systems concerning data governance and algorithm management. Understanding how data scientists and engineers create and evaluate algorithms is essential for healthcare professionals to engage in data management. Healthcare professionals must engage in conversations around data management if health systems want to be competitive in the health tech market. Training in data management for healthcare professionals, administrators, and patients is an important step to help create and maintain privacy standards and improve data sharing between organizations.

"How are we going to survive and make this transition into sort of a data-centric model, versus having all these silos where, you know, we're very protective of our data, but how do we engage with other organizations, how do we leverage the power of, um, data sharing in a way that maintains privacy?" EK

"AI now relies a lot on EHR data, but we are thinking about smartphones, wearable devices, huge amounts of data that patients are collecting, and patients are not sure they want to share that data. Do they want more transparency on how the data is being used?" AG

"I mean, when we go to a clinician and tell them, hey, here's a bunch of data, I mean, they're going to be interested for about six seconds, because they know that there's power in that, but it's sort of like taking a drowning man in the middle of the Atlantic and handing him a glass of water and saying, "here, this is going to be really good for you." RC

"If we do the work to be able to get information aggregated and accessible, will it actually be useful in a clinical setting? Will it actually improve outcomes?" RC

Process improvement 

Clinicians are drowning in data. How does an extensive data set show value in healthcare if a provider does not have the time or ability to analyze the data before them? Auto-summarization can pick out important pieces of a patient's entire chart, including structured and unstructured data, and synthesize them into an easily reviewable document for the provider. AI and machine learning (ML) can improve efficiency in back-office processes in billing, scheduling, and provider documentation. The improved efficiency of the process can decrease scheduling and billing errors leading to improved profit and patient experience. In addition, the improved efficiency in provider workflow can decrease documentation time, allowing the clinician more time with a patient.

"I think the low-hanging fruit, the problems that are solvable, the ones that are easy to measure, you know, are often financial, did I collect more revenue, can I get more patients seen in a day, can I get better utilization. Those are pretty discrete, right, and we can much more quickly and easily measure that" CF 

"I think we have a lot of people in healthcare who just don't want to deal with it and they put their head in the sand and say, 'no, I'm not going to do A.I. because I, no one can explain it,' and I think that that's a mistake, because we're missing an incredible opportunity. That would be like, you know, a hundred plus years ago and somebody says, 'yeah, you know what? I understand what you're talking about with germ theory of disease, but I'm not going to participate in that because I don't believe it. You can't show me a germ, I'm not going to believe it until you can.'" CN 

Physician experience 

Workflow-augmentation algorithms are being developed, utilizing natural language processing with ambient voice technology that decreases provider documentation time allowing for more time in the room with patients. ML and AI are being utilized to improve a provider's experience with chart documentation and data entry, allowing a provider to spend more time in critical thinking and providing care to patients during office visits and at the hospital's bedside. Increased provider documentation and data entry requirements are linked to an increased percentage of medical error and burnout. With the implementation of algorithms supporting workflow, a health system can improve quality measures of care and physician experience, leading to improved overall patient care. 

Knowledge augmentation is another area being explored through AI and ML methods. Companies are developing algorithms to improve quality and decrease error with the ultimate goal to improve outcomes. Chatbots have been developed and are being utilized as patient triage. Algorithms utilize large data sets and deep AI to learn how to read radiographs and allow providers to use diagnosis assist in EMR systems. AI is a tool, and knowledge augmentation is an area with great promise for deep AI algorithms to decrease variability in care with the ultimate goal of improving outcomes. The lack of explanation in how a deep AI algorithm produces a result is an ongoing concern for its use in medicine. Knowledge augmentation continues to be an avenue of research. 

"…. There's this other application, of A.I., around workflow augmentation. Taking things that are really burdensome, but relatively easy, and taking those off of people's plates, making that process a lot easier. And so we've seen that be very successful in other industries. I think it's been arguably even a more successful approach to the application of A.I. in non-healthcare, but healthcare continues to focus on the knowledge augmentation rather than workflow augmentation approach, relatively … 

So, if you can walk out of the room with your chart note already written, and ninety percent of your interview complete, and ninety-plus percent of your documentation complete … you're handing the clinician all of the information that they need in the most actionable, usable format possible. That is certainly an application of AI, to know what questions to ask, to know how to translate that information from the patient-friendly interview, into a chart-ready [provider] note." RC

The panelists discussed the importance of finding the right tool for a particular problem. In process improvement, finding the right tool involves understanding the problem compared to the end goal of success: quality improvement, physician experience, and patient outcome. Variability in healthcare leads to inconsistent care. Care should adhere to evidence-based guidelines and be consistent in quality and delivery. 

"I think a lot of us in healthcare are in it, yes to care for patients, but also to do it better, right, and we recognize in process improvement, we need to decrease the variability, right. The variation in care needs to get narrowed so that we can recognize if we are doing something right or wrong first, then we can correct it." CN

Value in data 

According to the 2021 Commonwealth Fund Report, the United States healthcare systems ranked last when compared in 71 measures, when rated against other industrialized nations globally [3]. Technology can add value to healthcare, but each program and algorithm must be strategically applied to the appropriate problem. Each organization must consider the ability to implement, maintain, and monitor AI and ML algorithms. Training must occur for clinical and administrative staff. The application and analysis of data sets are as or more important than the amount of data used. The ability of healthcare staff to understand results from algorithms and apply those results will determine the value of AI in mainstream medicine. RC compared value perceived by a consumer of social media and healthcare, saying, "… the interesting thing was it was not about building trust, it was about delivering value. And the truth is, Facebook was exciting and fun, and it let you connect with your friends and gave you a whole different way to be able to experience the Internet and interact, and it delivered on the value that people expected the same way as Google. I mean, I think that that's one of those places where healthcare has arguably failed in the past, and I think that that is arguably one of the roots of why we haven't seen more data sharing on the consumer side of things is because they don't see the value of making that data accessible."

"Look at 23andMe. I mean, people pay a hundred plus dollars for the right to give up their genomic information into an aggregate pool. Why'd they do it? Because they made it entertaining. It wasn't even a direct monetary value, in fact it was an inverse monetary value, you had to pay money for the right to get your information into that pool, and people just flowed in there because they made it engaging and entertaining." RC

"How do you see balancing the massive amounts of data that are out there, that need to be able to use it across some of those silos that have come up? But then, perhaps even more functionally, more importantly, is taking that data and turning it into information. Making it actionable, making it valuable." RC

Bias in healthcare and AI 

Bias in healthcare affects care pathways and processes in healthcare and has been an ongoing issue in medicine. AI algorithms highlight the bias already present in the system. An article by Igoe, titled “Algorithmic Bias in Healthcare Exacerbates Social Inequities: How to Prevent It,” highlights the algorithmic bias in healthcare [8]. The author discusses an example of racial bias in the Framingham risk study. In this study, nearly 80% of respondents were Caucasian. The bias of this study has the potential to affect outcomes when treating a diverse population [8]. In an article by Panch et al. (2019), titled “Artificial Intelligence and Algorithmic Bias: Implications for Health Systems,” the idea of inherent bias in algorithmic processes is discussed further [9]. The authors define algorithmic bias as the implementation of an algorithm that perpetuates the existing inequities in socioeconomic status, race, ethnic background, religion, gender, disability, or sexual orientation [9]. Panch et al. go on to describe the reality of this bias, “If the world looks a certain way, that will be reflected in the data, either directly or through proxies, and thus in the decisions” [9].

Data in healthcare have been biased due to the siloed nature of care the US healthcare system has created. Each organization owns its data, and the sharing of data between various groups is complicated. Populations served by various healthcare organizations may be homogenous, thus potentially creating a bias toward particular groups of people. Evaluating a group of people to note diagnosis and attempt to risk stratify the population is considered by the presenters as a way to help keep the focus of data on patient outcomes. Bias in data can lead to inconsistent care among different populations, and variability can lead to worse outcomes from a population standpoint.

"I want to maybe challenge everybody to think about it differently. It's actually exposing bias that's already there, right. We are already treating people differently, and we're getting away with it because, once again, they're in small scales that nobody has voiced it or it just hasn't been significant enough to be brought to their attention. But, when an algorithm does it, oh my god, everybody has to stop and turn off the algorithm and never use A.I. again." CN

"I’m excited that I think we’re going to find all kinds of different possibilities to take better care of our patients, but I think, maybe, to go even back to some of the earlier conversation, we need to gain not just the trust of the doctors on some of the algorithms and this, deep learning black box, you know, unexplainable A.I. We need to gain the trust of our patients too, to let us use the data, because insofar as we don’t have a kind of, heterogeneous population in our data, we’re going to have nothing but bias, and it’s going to exaggerate some of the inconsistent delivery of medicine that we have now.” CN

“I think what we need to do instead, if we can’t explain all of the nodes through deep learning and the machine learning component of it, we need to understand, what does that patient population look like, right? It’s more like a nutrition label, it contains this many of this kind of person, this many diabetics, this many hypertensives, this is their age, and apply it to a similar population to get the best results. And then what you have to do, as somebody using that algorithm, is you need to actually apply it to your population and let it run and make sure it’s giving you similar results before you turn it on and you have it start making recommendations in changing care whatsoever.” CN

## Discussion

**Figure 1:**
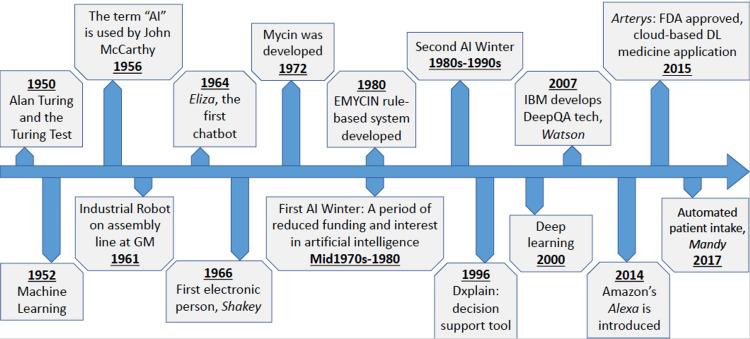
Timeline of artificial intelligence Adapted from Gastrointestinal Endoscopy Vol 92, issue 4, titled "History of Artificial Intelligence in Medicine”, figure adapted with permission from Elsevier 2020; copyright 2021 Gastrointestinal Endoscopy (receipt available upon request). AI: artificial intelligence, GM: general motors, DL: deep learning.

The term artificial intelligence was first coined in 1956 by John McCarthy. He defined AI as "the science and engineering of making intelligent machines” [10]. The field of AI is large and separated into different subfields including machine learning (ML), deep AI, and natural language processing (NLP). Machine learning works with established data sets and excels at pattern identification and analysis [11]. Deep AI or deep learning is composed of neural networks and allows the program or machine to learn and make autonomous decisions [11]. Natural language processing allows a machine to listen to a human voice and synthesize and analyze information based on conversations [11]. A detailed timeline of some aspects of the history of AI in medicine is shown in Figure 1, taken from an article in Gastrointestinal Endoscopy Vol 92, issue 4, titled "History of Artificial Intelligence in Medicine” [11]. The aforementioned panel consisted of five experts in their field all related to AI and data dissemination to improve processes and outcomes in healthcare. The symposium was designed as an accredited continuing medical education event. The panelists were asked to provide a 30-minute presentation and participate in an online moderated discussion. The moderated discussion centered around the various problems each participant felt AI is equipped to solve. AI is a tool, and one must figure out the problem before the implementation of the tool. Problems exist in medicine that are both process driven and provider/patient experience driven. AI, as a tool, has great promise to create improved pathways for scheduling, billing, and documentation within healthcare. AI can free up time for a provider to have improved conversation with patients, decrease errors in documentation, and potentially decrease burnout [12]. Currently, companies are using chatbots to assist in triage and funnel patients to the right provider. Some chatbots, such as one Babylon Health developed, even give treatment recommendations for low acuity issues [12]. In medical imaging, AI has been studied and shown to be as effective as specialists and radiologists in reading various image modalities across multiple specialties [6,7].

As highlighted by the panelists, knowledge augmentation is challenging to implement on a large scale in medicine. Great promise to add value to the provider’s decision-making process can be realized through AI algorithms, but it can also be a dangerous arena for the continuation of bias found in the sample data population used to create the algorithms as highlighted by the panelists. The discussion and exploration of knowledge augmentation algorithms will continue, and the potential for bias should not be taken lightly. Companies utilize AI in conjunction with EMR systems to improve a provider’s documentation time, make suggestions, and create notes using ambient voice technology, all within a workflow augmentation paradigm [7,13]. In one article, ambient voice technology with natural language processing may forever alter the way a provider documents, allowing for more time with the patient and less time on chart documentation [13]. Barriers to implementation include upfront cost, training, data sharing, and compatibility with the existing technology [13,14]. The authors also discussed bias and risk to data privacy as potential issues to be aware of [13]. True knowledge augmentation algorithms should be developed without bias and meticulously follow evidence-based diagrams. The algorithms should be explainable to the provider, meaning that the steps taken to reach a diagnosis should be discoverable. As alluded by the participants, if an algorithm makes medical decisions, it should go through board certification. 

Workflow augmentation is perhaps a more promising area for AI to provide meaningful assistance to problem areas in billing, scheduling, and nursing workflows and triage. AI performs faster and more efficiently than humans in billing, scheduling, accounting, and management of processes. AI chatbots can improve efficiency in nursing and triage processes in the clinic [15]. Healthcare is a complex system of professionals, departments, and institutions. Utilizing engineer planning paradigms such as Reach, Effectiveness, Adoption, Implementation, and Maintenance (RE-AIM) and Systems Engineering Initiative for Patient Safety (SEIPS) to evaluate, implement, and monitor AI-enabled systems within the culture of each department the system interacts with provides an opportunity to collect data on how AI has addressed a given process and decreased inefficiencies [16]. Instead of looking just at the tool's effectiveness, Li et al. suggest the importance of understanding the workflows of each individual department and how this will change the utilization of AI [16]. The entire system including workflows altered or vanquished is evaluated as well as the effect of the particular algorithm on quality and efficiency [16]. The delivery science rests on three principles: healthcare is delivered in complex systems and AI must adapt; AI should be viewed as a tool to be used in part of the broader system and not as the end product; and the problems that AI addresses consist of a complex web of people, process, and technologies [16]. The implementation of AI and ML in medicine should not be about implementing a particular algorithm. Instead, quality healthcare systems are built by finding the best solution available to each particular set of problems [16]. Finding the right tool for the right problem is essential to consider when evaluating the implementation of AI in healthcare. For example, the authors discuss an algorithm built to predict acute kidney injury with high accuracy [16]. This was built under the premise that the algorithm would improve care by decreasing time to diagnosis. However, when put into practice, the algorithm was a burden to physicians and value to the provider and patient was determined to be unclear [16]. This example highlights the importance of understanding the workflows the algorithm will affect [16]. Building an algorithm for the sole purpose of predicting a task in healthcare is not adequate to improve care. The broad view of all persons, departments, and workflows affected must be taken into account [16]. 

Data privacy and access is a critical issue and one in which policy and process must be developed to ensure the safe acquisition and transfer of data in the healthcare sector. The exchange of patient data among healthcare institutions, academic research, and industry can declassify sensitive information and allow malicious data breaches [17]. Efforts must be taken to limit the risk of such data breaches. Before using an extensive data set of patient information, understanding issues involving patient consent is essential [17]. Some ethics committees do not require consent for deidentified information, while some prefer to use an opt-out method. This method could lead to bias as only the most engaged patients will be involved [17]. Value is an essential factor to consider when implementing a new product or service. The same is true for healthcare and especially AI in healthcare. For a system or provider to change workflow, the value of the change to the new service must be shown. The user and the consumer must see value in the service and gain trust to utilize the service. The perceived risks to the implementation of AI include fear of new technology, ethical or trust issues, and regulatory concerns [18]. Performance and communication concerns were noted to be the highest predictor of risk belief among survey participants [18]. The panelists discussed the data and the reality of data fatigue for the healthcare provider. The analysis of data should be meaningful for the endpoint of a particular problem. Brault and Saxena discussed the large amounts of data collected with mobile health technology and questioned the validity of the data due to inherent bias [19]. The authors suggest the value of data is not just the amount collected but the appropriate evaluation and analysis of said data [19]. 

Unconscious bias in healthcare is understood as the attitude and opinion toward a particular person or group that affects perceptions by changing the way the care is provided [20]. Bias is inherent in the way medicine is practiced. The siloed nature of care in the United States creates homogenous pools of patients that data are pulled from research and process improvement projects. Algorithms expose bias already embedded in the process due to the particular data set being studied, a theme that emerged in the discussion. Brault and Saxena offer three principles to keep in mind for further research. The first principle is to create a catalog of bias listing the source and ramifications of bias [19]. The second deals with creating standards for the use of AI as a tool in medicine [19]. The third principle the authors discuss concerning the research of AI is to develop an approach to evaluate and analyze the effectiveness of AI to solve a particular problem [19]. The reality of AI and the change to specific processes and workflows in medicine will require training for healthcare professionals in the basic language of AI and algorithms. Training will open up the world of AI to professionals and help to safeguard this growing technology in the complex arena of medicine.

Limitations 

Although approximately 50 people registered for the event, less than half were able to attend. Reasons for this could be online meeting fatigue, time of day, and lack of good reminders. Although the event had technical difficulties initially and the first five minutes was not captured, 52 minutes of discussion was transcribed and analyzed. The symposium drew expert panelists from various aspects of the health technology industry; however, more industry leaders should be included in this discussion, including, but not limited to, experts on interoperability, health policy as it relates to AI and emerging technology, and system strategy and management to evaluate new care models emerging through the use of AI.

The research for the analysis was conducted through Google Scholar. The search could have been expanded to include other search engines to potentially include more articles for study. Words such as machine learning, deep AI, and neural networks were uncovered with the search conducted; however, the search could be completed using these words and others to potentially increase the article pool.

## Conclusions

The panel discussion highlighted several issues to consider when thinking about the implementation of AI into medicine. Data privacy and access is a critical issue and one in which policy and process must be developed to ensure the safe acquisition and transfer of data in the healthcare sector. Many areas will need continued discussion and research for the successful implementation of AI in medicine, including a continued look into workflows and how AI can add value and a look at various ways AI can add value with knowledge augmentation. AI algorithms highlight bias that exists within health system processes and policy. Algorithms have the potential to shed light on areas that need improvement to decrease bias and improve equity in care among all people regardless of race, gender, and socioeconomic status. More research is needed in the area of value and bias of AI in a healthcare setting. Standards for the implementation of AI and a methodical approach to the analysis of the effectiveness of algorithms coupled with training of healthcare professionals in the language of AI algorithms will be helpful to integrate AI into mainstream medicine safely.
